# *Beauveria bassiana* Keratitis: A Case Series and Review of Literature

**DOI:** 10.3390/jcm12247601

**Published:** 2023-12-10

**Authors:** Karl Anders Knutsson, Paolo Rama, Beatrice Tombolini, Carlo Di Biase, Carlotta Senni, Fabio Buffoli, Nicola Clementi, Marco Tonelli, Silvia Carletti, Nicasio Mancini, Giulio Ferrari, Giorgio Paganoni, Francesco Bandello

**Affiliations:** 1 Department of Ophthalmology, IRCCS San Raffaele Scientific Institute, 20132 Milan, Italy; tombolini.beatrice@hsr.it (B.T.); senni.carlotta@hsr.it (C.S.); ferrari.giulio@hsr.it (G.F.); paganoni.giorgio@hsr.it (G.P.); bandello.francesco@hsr.it (F.B.); 2 University Eye Clinic, Fondazione IRCCS Policlinico San Matteo, 27100 Pavia, Italy; p.rama@smatteo.pv.it (P.R.); c.dibiase@smatteo.pv.it (C.D.B.); 3 Laboratory of Medical Microbiology and Virology, University Vita-Salute San Raffaele, 20132 Milan, Italy; buffoli.fabio@hsr.it (F.B.); clementi.nicola@hsr.it (N.C.); carletti.silvia@hsr.it (S.C.); 4 Laboratory of Medical Microbiology and Virology, IRCCS San Raffaele Scientific Institute, 20132 Milan, Italy; tonelli.marco@hsr.it; 5 Laboratory of Medical Microbiology and Virology, University of Insubria, 21100 Varese, Italy; nicasio.mancini@uninsubria.it

**Keywords:** *Beauveria bassiana*, fungal keratitis, cornea, in vivo confocal microscopy, infectious keratitis

## Abstract

Background: *Beauveria bassiana* is a filamentous fungus commonly used as an insecticide that rarely causes keratitis. Methods: Patients affected by *Beauveria bassiana* keratitis were retrospectively recruited at San Raffaele Hospital (Milan, Italy) between 2020 and 2022. All subjects underwent comprehensive ophthalmic evaluation, including in vivo confocal microscopy (IVCM) and microbiologic examination of corneal scrapings. *Beauveria bassiana* was identified using 18S rDNA targeted PCR. Results: Four eyes of four patients (51 ± 8.8 years old) were evaluated. The main risk factors were soft contact lens wear (75%) and trauma with vegetative matter (50%). A superficial infiltrate was displayed in the majority of patients. Three cases (75%) showed hyphae on IVCM. All patients showed clinical improvement after topical antifungal therapy, although mostly through a combination of two antifungals (75%). One patient with a deeper infection required a systemic antifungal agent after one month of topical therapy. All cases required debridement to reduce the microbial load and enhance drug penetration. All patients experienced keratitis resolution following medical treatment (average: 3.3 months). Conclusions: The identification of risk factors and the early diagnosis of *Beauveria bassiana* keratitis are fundamental in order to avoid its penetration in the deeper corneal stromal layers. Topical antifungal drugs, possibly accompanied by ulcer debridement, may be a successful treatment if instilled from the early phases of the disease.

## 1. Introduction

Fungal keratitis (FK) is an important cause of blindness, particularly in regions with a tropical climate [1]. The incidence of FK, as well as the species involved, vary largely, even within different regional areas of the same nation. The reported incidence of FK is 34.4% in South India [2], 35% in southern Florida [3], and 37.5% in Ghana [4], with lower values in temperate areas such as New York and London (1–5%) [5,6]. These data were obtained from tertiary referral centers. Specifically, the South India study [2] involved two centers over a 3-year period (1999–2002), the Florida study [3] involved a single center over a 9-year period (1969–1977), the study based in Ghana [4] involved a single center with an unknown time frame, the New York study [5] involved a single center in a 17-year period (1987–2003), and the London study [6] involved a single center over a 13-year period (1993–2007). These variations are related not only to climate, but also to gender, age, socioeconomic setting, agricultural work, and degree of industrialization [1,7]. In particular, a lower socioeconomic status may be related to reduced hygiene and health education, whereas agricultural work and a lower degree of industrialization are likely linked to increased activity in open rural areas, where eye trauma is more likely.

The local predisposing factors for the development of FK are trauma (principally with vegetative matter), contact lens use, and chronic therapy with topical steroids [1,8]. Specifically, microtrauma with vegetable matter was present in 55–65% of cases of fungal keratitis in large Indian series [9,10,11] whereas contact lens wear is a more significant risk factor in industrialized countries. [12] For example, a French series [12] reported an increase of contact-lens-associated infection from 35.5% to 71% when comparing two different time periods (1993–2008 vs. 2014–2018). Topical steroids are considered to be a principal factor for the development of fungal keratitis [8]. Nevertheless, the percentages are notably variable with reported steroid use as the initial therapy in 1–60% of subjects [10,13,14,15,16,17].

Other significant risk factors include farming activity, diabetes, human immunodeficiency virus infection, ocular surface pathology, previous corneal transplantation and ocular surgery, exposure keratitis, and herpes simplex virus keratitis [7].

*Beauveria bassiana* is a filamentous fungus of the Ascomycota division which is found ubiquitously in soil and other substrates. Because of its entomopathogenic characteristics, it is used as an insecticide and rarely causes infections in humans [18]. *Beauveria bassiana* has been isolated in opportunistic lung and tissue infections in immunocompromised patients [19,20,21] and in a few reported cases of fungal keratitis [18,22,23,24,25,26,27,28,29,30,31,32,33,34,35,36,37].

In this case-series study, we presented four cases of *Beauveria bassiana* keratitis and reviewed the current available literature regarding the management of this pathogen. Specifically, we analyzed the risk factors, treatment regimens utilized, and final outcomes, including requirement of surgical treatment. The aim of the study is to share our clinical experience in a tertiary referral center with the availability of confocal microscopy and commonly used antifungal agents, which were most often utilized in combination therapy. *Beauveria bassiana* keratitis is a rare entity with less than twenty cases reported in literature, and, to the best of our knowledge, this represents the largest case series reported in literature.

## 2. Methods

This was a case series of consecutive patients affected by *Beauveria bassiana* keratitis. This study was retrospectively conducted at the Cornea and Ocular Surface Disease Unit, San Raffaele Hospital (Milan, Italy) between 2020 and 2022. All subjects underwent comprehensive ophthalmic evaluation, including in vivo confocal microscopy (IVCM) and microbiologic examination of corneal scrapings. The study followed the tenets of the Declaration of Helsinki (1975) and its following amendments. In light of the retrospective nature of the current study, ethics approval by the local Istitutional Review Board was not required. The diagnostic therapeutic management belonged to standard medical procedures; no experimental treatments were utilized. Detailed patient history was taken to allow identification of possible risk factors. Written consent to publish potentially identifying information including details of the case and photographs was obtained by all patients after reading and signing specific forms. All authors attest that they meet the current ICMJE criteria for authorship.

The review of literature was performed by searching the electronic databases PubMed^®^, and Ovid Medline using search terms related to *Beauveria bassiana* fungal keratitis. The following search terms were utilized: “*Beauveria bassiana* keratitis”; “*Beauveria bassiana* ocular”; and “*Beauveria bassiana* eye”. Additional studies were identified by manually searching the reference list of the included studies. The electronic database search was conducted in September 2023.

As for corneal scrapings, they were inoculated by the ophthalmologist on chocolate agar (PVX, Biomerieux, Marcy l’Etoile, France), on Sabouraud agar (SGC2, Biomerieux, Marcy l’Etoile, France), and on a homemade plate of Nonnutrient Agar (NA) supplemented with *E. coli* for *Acanthamoeba* spp. culture. Two scraping smears were also made and Gram and Calcofluor white stains (CFW) were performed. Lactophenol-cotton blue (LPCB) tape mount was prepared after significant growth of mold colonies.

Then, 18S sequencing was used to confirm mold identification. In the details, fungal colonies were collected and lysed by using QIAamp DNA Mini Kt (QIAGEN): buffer ATL solution was supplemented with K proteinase (Qiagen, Vienna, Austria) and sample was vortexed at 500 rpm and heated at 90 °C for at least one hour. Then, AL buffer was added and the sample was further heated at 95 °C for 15 min. DNA was then extracted by using ELITe InGenius^®^ (Elitech, Turin, Italy) following manufacturer instructions. DNA extract was then amplified for generating 18s rDNA amplicon. Amplification was checked through agarose gel-electrophoresis. Extraction, as well as amplification, positive and negative controls were added on both amplification reaction and electrophoresis [38]. The 18S amplicons were then sequenced trough Sanger sequencing and sequence electropherograms were elaborated by using SeqScape informatics suite. Consensus sequence was analyzed on both NCBI Blast (https://blast.ncbi.nlm.nih.gov/, accessed on 1 June, 2023) and Mycobank (https://www.mycobank.org/Pairwise_alignment, accessed on 1 June, 2023) for inferring fungal databases.

## 3. Case Series

Four cases of four patients (51 ± 8.8 years old) affected by *Beauveria bassiana* keratitis were described. No patients had an immunocompromised status at the onset of infection. The main risk factors were soft contact lens wear (75%) and trauma with vegetative matter (50%). The majority of patients displayed a superficial infiltrate. Three cases (75%) showed hyphae on IVCM. All cases reported a clinical resolution of keratitis after a short topical medical treatment (3 ± 0.8 months), although a combination of two antifungals was necessary in three cases (75%). One patient (25%) with a deeper infection required a systemic antifungal agent after one month of topical therapy. Debridement was performed in all cases to reduce the microbial load and enhance drug penetration. The clinical features, therapeutic management, and evolution of each case are summarized in Table 1.

### 3.1. Case 1

**Figure 1:**
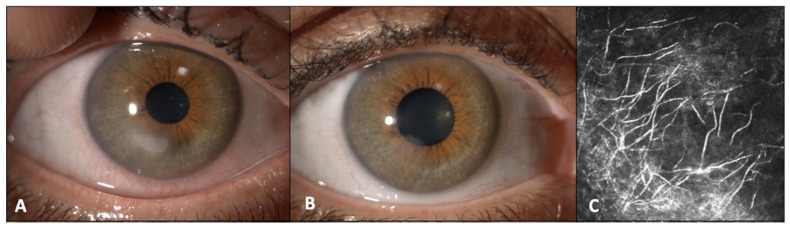
Clinical presentations and confocal microscopy of corneal infiltrate in Case 1 (**A**–**C**). (**A**) Initial presentation of the peripheral corneal infiltrate caused by *Beauveria bassiana.* (**B**) Resolution of the corneal infiltrate in Case 1 after three months of topical therapy with 1% voriconazole. (**C**) Confocal microscopy of the corneal infiltrate, revealing presence of hyperintense branching lines compatible with presence of hyphae in the anterior corneal stroma.

A 57-year-old woman was referred to our department for the evaluation of a contact-lens-related keratitis that had started three weeks before. The patient had been attending regular follow-up visits for a past posterior optic neuritis that recovered with good residual visual acuity. At the time of the first visit, she was under treatment with topical steroids six times daily and topical tobramycin every two hours without improvement. The right eye was painful and the best corrected visual acuity (BCVA) was 20/200. On a slit lamp examination, a peripheral infiltrate with an associated epithelial defect, approximately 1 × 1 mm in size, was observed (Figure 1A). No associated corneal hypoesthesia was reported. The concomitant finding of hyphae during the IVCM examination suggested a fungal etiology (Figure 1B). Corneal scraping was performed and sent to the microbiology laboratory. Gram stain showed many polymorphonuclear leukocytes (PMNs), while CFW was negative. Empirical treatment with hourly cefazolin 5%, hourly tobramycin 2%, hourly voriconazole 1%, and bedtime ofloxacin 0.3% ointment was initiated. Initially, the infiltrate slightly increased in size (2 × 1 mm), most likely due to the discontinuation of topical steroids. However, in the following visits, an objective improvement was observed as demonstrated by the reduced density and the better-defined margins. Debridement of the infiltrate was regularly performed to reduce the microbial load and adjuvate drug penetration. After eight days, the corneal scraping culture was found to be positive for white cottony mold colonies on Sabouraud agar. LPCB tape mount showed wide septate hyphae with little or no conidiogenous cells, which made it challenging to differentiate the strain from other hyaline fungi. *Beauveria bassiana* was identified by 18S sequencing. The patient continued topical voriconazole therapy for over 3 months, until a complete resolution of the keratitis was achieved (Figure 1C).

### 3.2. Case 2

A 59-year-old woman was referred to our department for a contact-lens-related keratitis of recent diagnosis. At the time of our evaluation, the woman was under treatment with hourly topical moxifloxacin, hourly tobramycin, and bedtime tobramycin ointment. A slit lamp examination revealed an inflamed eye with a central, poorly defined, superficial infiltrate and two endothelial deposits. Vision was counting fingers. The patient disclosed accidental contact with vegetative matter after cutting ginger root during cooking activity, that occurred two days prior to the development of symptoms. IVCM showed the presence of hyphae, suggesting a fungal etiology. Corneal scraping was performed and sent to the microbiology laboratory, which confirmed the presence of hyphae with CFW. Empirical topical therapy with hourly amphotericin B 0.3%, hourly voriconazole 1%, and moxifloxacin 0.5% six times daily was prescribed. Following the onset of therapy, the infiltrate borders became more defined, suggesting control of the underlying infection. However, a concomitant increase of inflammation was observed, with the appearance of hypopyon, descemetic folds, and an increased number of endothelial deposits which, subsequently, coalesced into an endothelial plaque. After nine days, mold colonies grew on Sabouraud agar and the LPCB microscopic features showed an abundance of sterile hyphae and rare or no significant structures. Four days later, the corneal scraping was found to be positive for *Beauveria bassiana*, following identification by 18S sequencing. The patient remained on topical therapy with hourly voriconazole 1% and amphotericin B 0.3% eight times daily for over a month while the antibiotic was slowly tapered. IVCM was repeated and revealed a reduction in hyphae, which were still present in the deeper stromal layers. Systemic itraconazole, 100 mg two times daily was prescribed after one month, for a total of 30 days. Keratitis slowly improved: the infiltrate became more superficial and better defined, and infiltrate debridement was regularly performed to reduce the microbial load and promote drug penetration. After approximately two months of therapy, topical dexamethasone 0.1% (one drop every three days) was added to improve the underlying inflammation. After four months of therapy, fungal keratitis completely resolved leaving a corneal opacity and vision remaining as counting fingers.

### 3.3. Case 3

**Figure 2:**
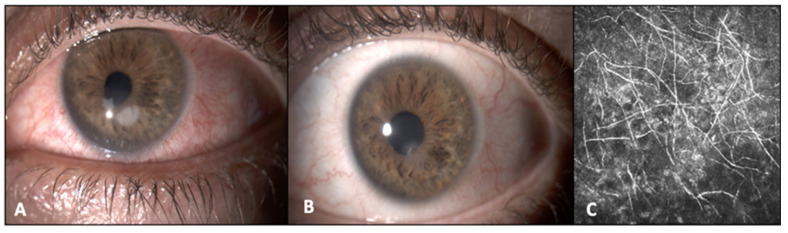
Clinical presentations and confocal microscopy of corneal infiltrate in Case 3 (**A**–**C**). (**A**) Initial presentation of corneal infiltrate caused by *Beauveria bassiana*. (**B**) Resolution of the corneal infiltrate in Case 3 after 2.5 months of therapy with two topical antifungal drugs (1% voriconazole and 5% natamycin). (**C**) Confocal microscopy of the corneal infiltrate. Structures compatible with hyphae can be identified in the anterior corneal stroma.

A 40-year-old man presented with a contact-lens-related, long-standing keratitis of suspected infectious etiology that had started 4 months prior to our evaluation. At the time of our visit, he was under treatment with topical ofloxacin four times daily, topical ganciclovir three times daily, and topical dexamethasone two times daily. The patient fortunately had been maintaining a good BCVA (20/20). Under a slit-lamp biomicroscopic evaluation, corneal hypoesthesia, and a corneal infiltrate 1.5 × 1.5 mm with feathery margins and an associated epithelial defect were noted (Figure 2A). Corneal scraping was performed and sent to the microbiology laboratory. Only a Gram stain was prepared due to the lack of material and showed many PMNs with septate hyphae. IVCM, which was also performed, revealed the presence of hyphae (Figure 2B). A topical therapy with cloramphenicol/tetracycline association every 2 h and hourly voriconazole 1% was initiated. Hourly topical natamycin 5% was added after one week, since infiltrate size remained stable without clear signs of improvement. Debridement was performed during the following follow-up visits. After seven days, Sabouraud agar was positive with white floccose mold colonies. *Beauveria bassiana* was identified by 18S sequencing, since the LPCB tape mount showed no significant microscopic fungal structures. The patient continued therapy with voriconazole, natamycin, and cloramphenicol/tetracycline association. Therapy was slowly tapered over the following two months, as the infiltrate kept improving, and was stopped once the eye became quiet, resulting in a superficial corneal leukoma (Figure 2C).

### 3.4. Case 4

A 48-year-old man presented to our department with keratitis that had begun about a month before our evaluation, following trauma with an olive tree branch. He was under treatment with topical levofloxacin six times daily, topical chloramphenicol six times daily, voriconazole 1% eye drops six times daily, and systemic fluconazole 100 mg daily, with no improvement. He previously underwent a corneal scraping that tested negative for bacteria and fungi. Clinical examination revealed a 2 × 2 mm paracentral corneal infiltrate with irregular margins and a 0.5 × 0.5 mm epithelial defect. Iridolenticular synechiae were also observed. Vision was 20/20, uncorrected. Corneal scraping was performed and sent to the microbiology laboratory. Both the Gram stain and CFW showed septate hyphae. IVCM was also performed but did not reveal the presence of fungal structures. Therapy was stopped for 24 h before performing corneal scraping.

**Figure 3:**
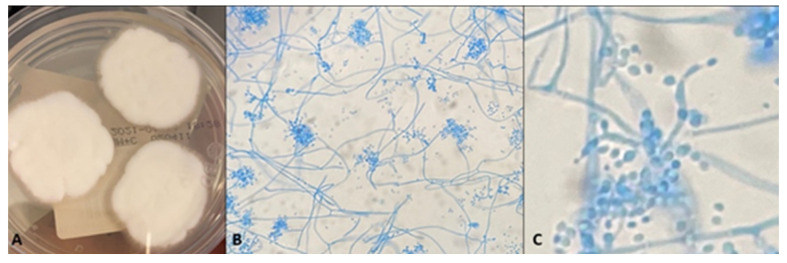
Cultural examination in Case 4 (**A**–**C**). (**A**) Typical white floccose colonies of *Beauveria bassiana* on Sabouraud dextrose agar. (**B**) LPCB slide mount showing sympodial development of conidiogenous cells forming dense clusters at 400× magnification. (**C**) Details of flask-shaped, geniculate conidiogenous cells with ovalar conidia (3.5× digital zoom on top of 400× magnification).

Empirical treatment with hourly amphotericin B 0.3%, hourly voriconazole 1%, and hourly levofloxacin 0.5% was initiated and oral medication was stopped. Debridement of necrotic tissue was regularly performed. Ten days later, typical floccose white thick colonies grew on Sabouraud agar (Figure 3A). LPCB tape mount was performed and showed many clusters of conidiogenous cells and loose conidia which were not evidenced in previous cases, as well as wide hyphae (Figure 3B,C). Based on previous experience and on the macroscopic and microscopic features of the mold, *Beauveria bassiana* was identified.

The following examinations revealed that the infiltrate was gradually resolving, with the resolution of the epithelial defect. The infiltrate resolved after three months of treatment, and the patient retained 20/20 vision.

## 4. Discussion

Entomopathogenic fungi are constituents of terrestrial ecosystems and are involved in the dynamics of arthropod populations in both natural and agricultural ecosystems [39,40]. These micro-organisms are currently used in agriculture as low-environmental-impact pesticides. Recently, their use has increased, and the most commercialized products are based on *Beauveria* spp. and *Metarhizium* spp. [40].

The main risk factors for the development of fungal keratitis are trauma (principally with vegetative matter), contact lens use, and chronic therapy with topical steroids [1]. The early diagnosis of fungal keratitis is complex, since the characteristics of the lesions at the onset of symptoms are not pathognomonic. Moreover, FK may be mistaken for other types of corneal infection, although their symptoms are usually more durable (5–10 days) and their onset is as not rapid as bacterial keratitis [41]. It has been reported that only 45% of cases of fungal keratitis are appropriately identified on clinical examination [42]. For these reasons, corneal scraping and microbiological investigation are fundamental and ideally should be performed before initiating specific antimicrobial therapy. Metagenomic deep sequencing is a novel approach that has been proposed to improve diagnostic sensitivity and accuracy in the management of infectious keratitis [43]. A recent study [44] has confirmed its potential, demonstrating a higher sensitivity and specificity of the metagenomic approach with RNA sequencing compared to standard KOH/Gram stains and cultures.

*Beauveria bassiana* keratitis has likely increased in frequency considering that the rate of published reports has risen dramatically in the past 30 years, possibly related to its use in agriculture. The first report of keratitis related to this pathogen was in 1984, but approximately 50% of cases have been described in the last decade only. In Table 2, we summarized the main features of 18 cases previously reported in literature.

The majority of these patients were immunocompetent, although, in six cases (33.3%), some degree of immunosuppression was present because of diabetes mellitus or topical corticosteroid treatment. In almost all patients, the risk factors for infection were identified: three cases were post-traumatic, three cases were in soft contact lens users, five cases were in farm or agricultural workers, two patients lived in remote rural areas, two patients had undergone previous keratoplasty (one had corneal endothelial transplantation for Fuchs’ dystrophy, while the other had penetrating keratoplasty for keratoconus), one case had bullous keratopathy, and one case was affected by Fuchs’ corneal dystrophy. With regard to immune status, three patients were affected by diabetes, two were utilizing topical steroids, and one was both diabetic and utilizing topical steroids. In two cases, the risk factors were unknown. Before a clear diagnosis of fungal keratitis and before starting antifungal drugs, 13 out of 18 patients (72.2%) had received treatment with topical steroids. Overall, treatment was successful in 16 of 18 patients (88.8%), and 12 (66.7%) of these patients required surgical treatment, including penetrating keratoplasty, superficial keratectomy, ulcer debridement, amniotic membrane transplantation, deep lamellar dissection, and vitrectomy. When analyzing the cases that did not recover vision, one eye (0.5%) was eviscerated directly due to infection, whereas another eye (0.5%) did not recover due to total retinal detachment after an emergency therapeutic keratoplasty.

The risk factors of the present case series were soft contact lens use in three patients (75%), and trauma with vegetative matter in one patient (25%). One patient also had a non-traumatic accidental exposure to ginger root fragments during cooking. This root was cultivated in a pesticide-free environment, where entomopathogenic fungi including *Beauveria bassiana* were regularly used, according to the agricultural company responsible for production. However, the clinical relevance of this risk factor is not clear, as the patient also wore contact lenses, which alone represents a risk factor for the development of keratitis. No patients had an immunocompromised status at the onset of infection. In two cases, topical steroids had been initiated before reaching our attention and before the diagnosis of *Beauveria bassiana* keratitis.

All cases responded well to topical antifungal therapy and one patient also received a systemic antifungal agent. In all cases, debridement of the necrotic tissue and epithelium was regularly performed with a Kimura-like spatula. The role of debridement has already been described in literature and allows a reduction in the microbial load and an increase in drug penetration [45,46]. Different authors report using adjunct epithelial debridement to treat *Beauveria bassiana* keratitis [25,30,31,32]. In our opinion, debridement is particularly advantageous in this infection due to the slow growth of the pathogen and limited tissue inflammation. However, in some cases, debridement may be contraindicated due to the decreased corneal thickness and risk of perforation. In all cases, after corneal scraping was performed, empirical antibiotic therapy was continued, as bacterial superinfection could not be excluded. Since the final culture results may arrive after more than a week, we were forced to rely on empirical treatment.

In more detail, the first case responded well to voriconazole and debridement. In the second case, a dual antimycotic treatment was immediately initiated (amphothericin B and voriconazole) due to the presence of hyphae in the deep corneal stroma identified by confocal microscopy and due to the large infiltrate size and concomitant inflammation. Systemic itraconazole was initiated after one month since confocal microscopy still revealed the presence of hyphae in the deep stromal layers. Itraconazole was chosen based on previous reports of efficacy in cases of severe *Beauveria bassiana* keratitis [18,27]. In the third case, there was no initial response to voriconazole, and we obtained successful control of the infection only after adding a second antimycotic agent, natamycin. In the fourth case, the patient was already undergoing antifungal therapy with topical voriconazole and systemic fluconazole without a sufficient clinical response. A dual therapy with amphothericin B and voriconazole proved to be successful.

From the analysis of previous case reports, *Beauveria bassiana* appears to be relatively resistant to antifungal agents. In most cases, however, topical and systemic antifungal therapy was successful in treating infection, especially when natamycin or azoles (voriconazole or itraconazole) were used [25,27]. We confirm these findings with a high rate of therapeutic success. Even though antimicograms were not available, we noticed clinical resistance to one antifungal agent in most cases, which required the prescription of a second topical antifungal agent. In our experience, we observed a low aggressiveness of the pathogen, compared to other fungal infections. Previous literature has described the corneal invasion by *Beauveria bassiana* as frequently constrained to the surface of the cornea, in contrast to other filamentous fungi, such as *Aspergillus* spp. or *Fusarium* spp., which can cause a deeper stromal invasion generally accompanied by severe anterior chamber inflammation [27]. In an experimental rabbit model, *Beauveria bassiana* grew slowly in corneal cells, confirming a low capacity of extension into the anterior chamber [47]. Some authors postulated that this behavior is due to its inability to grow at 35–37 °C [30]. Even though we obtained clinical improvement through topical antifungal therapy in all our cases, we must consider that, in three cases (75%), the corneal infiltrate was superficial. One patient (25%) with a deeper infection received a systemic antifungal agent, due to the persistence of hyphae in the deep stroma after one month of topical therapy on IVCM. This patient responded well to topical therapy alone, but adding a systemic agent was preferred in order to reduce the possible risk of anterior chamber invasion. It is possible that deeper infections may be harder to treat, requiring more invasive approaches, such as systemic medications, keratectomy, or therapeutic penetrating keratoplasty.

The current study showed some drawbacks, mainly due to its small-sized population, single-center setting, and the absence of antimicograms, which would have been useful to correlate with cases of clinical resistance to specific antifungal agents. Furthermore, a whole genome analysis was not available for a comparison of the isolated strains to the strains used for agricultural purposes.

## 5. Conclusions

Since entomopathogenic fungi are more often used in the agricultural world, these emerging pathogens could be more frequent in the next decades. The identification of risk factors and the early diagnosis of *Beauveria bassiana* are fundamental in order to avoid the penetration of these fungi in the deep corneal stroma. Although this filamentous fungus appears to be relatively resistant to antifungal agents, in most cases, topical antifungal therapy was successful in treating infection, especially if initiated in the early phases of the disease. In the case of deeper infections, more invasive approaches, including systemic antifungal medications, keratectomy, or therapeutic penetrating keratoplasty, may be required.

## Figures and Tables

**Table 1:** Clinical, therapeutical management, and clinical evolution of four cases of patients affected by *Beauveria bassiana* keratitis.

	Risk Factors	Time of Onset of Keratitis (Days)	Initial Medical Treatment	IVCM	Time for Positive Culture (Days)	BCVA at Diagnosis	Pharmacological Treatment after Diagnosis of Fungal Keratitis	Clinical Evolution
Case 1 (female, 57 yo)	Contact lens	21	- Betamethasone 0.13% + Chloramphenicol 0.25% drops - Tobramycin 0.3% drops	+	8	20/200	- Voriconazole 1% drops - Cefazolin 5% drops - Gentamicin 2% drops - Ofloxacin 0.3% ointment	Good response to topical voriconazole and debridement. Final BCVA: 20/25 Follow-up: 1 year
Case 2 (female, 59 yo)	Contact lens Accidental contact with vegetative matter during cooking	7	- Moxiflocaxin 0.5% drops - Tobramycin 0.3% drops - Tobramycin 0.3% ointment	+	9	Counting fingers	- Voriconazole 1% drops - Amphothericin B 0.3% drops - Moxifloxacin 0.5% drops - Itraconazole 100 mg twice daily (tablets) - Dexamethasone 0.1% (after two months)	Severe rapid onset keratitis. Good response to a combination of two topical antifungal drugs. Systemic Itraconazole was added for one month to reduce dissemination to anterior chamber. Dexamethasone 0.1% (one drop every 3 days) was initiated to control inflammation. Debridement was performed. Keratitis resolved after 4 months of therapy. Final BCVA: counting fingers Follow-up: 1 year
Case 3 (male, 40 yo)	Contact lens	122	- Ofloxacin 0.3% drops - Ganciclovir 0.15% gel - Dexamethasone 0.1% drops	+	7	20/20	- Voriconazole 1% drops - Chloramphenicol 0.4% + Sodium colistimethate 180.000 UI/mL + Tetracyclin 0.42% drops - Natamycin 5% drops	Poor response to voriconazole and antibiotic combination drops. Improved with the addition of natamycin. Debridement was performed. Final BCVA: 20/20 Follow-up: 6 months
Case 4 (male, 48 yo)	Trauma (tree branch)	31	- Levofloxacin 0.5% drops - Chloramphenicol 0.5% drops - Voriconazole 1% drops - Fluconazole 100 mg once daily (tablets)	−	10	20/20	- Voriconazole 1% drops - Amphothericin B 0.3% drops - Levofloxacin 0.5% drops	Good response to a combination of two topical antifungal drugs. Debridement was performed. Final BCVA: 20/20 Follow-up: 6 months

Abbreviation: yo: years old; IVCM: in vivo confocal microscopy; BCVA: best corrected visual acuity.

**Table 2:** Main features of the eighteen cases of *Beauveria bassiana* keratitis previously described in literature (1984–2021).

Ref	Year	Country	Age, Sex	Immune Status	Risk Factor	Antifungical Treatment	Steroid before Antifungical Treatment	Surgical Treatment	Outcome
[22]	1984	Japan	44, M	Competent	Unknown	Endovenous: M	Yes	None	Recovered (VA 20/20)
[23]	1985	USA (Massachusetts)	64, M	Competent	Trauma	None	Yes	PK	Recovered (PK after corneal perforation)
[24]	1997	Australia	67, F	Competent	Unknown	Topical: M, N	Yes	Lamellar dissection	Recovered (VA 20/30)
[25]	2000	USA (Illinois)	82, F	Competent	Trauma, Fuchs’ corneal endothelial dystrophy	Oral: F Topical: N	Yes	PK	Recovered (VA 2/60)
[26]	2007	USA (Illinois)	58, F	Competent	Soft contact lens wearer	Oral: F Topical: N, P, V	Yes	None	Recovered
[27]	2008	Japan	80, F	Compromised (diabetes mellitus)	Trauma	Oral: I Topical: M, N, V	Yes	None	Recovered
[28]	2008	South Korea	62, F	Competent	Trauma, farm worker	Oral: I Intravitreal/topical: A	Yes	PPV, evisceration	Unrecovered
[29]	2008	USA (New Mexico)	55, F	Compromised (diabetes mellitus)	Unknown	Oral: K, V Topical: K, N	Yes	PK	Recovered (VA 20/20)
[29]	2008	USA (Wisconsin)	31, F	Competent	Soft contact lens wearer	Oral: V Topical: A, N	Yes	None	Recovered (VA 20/25)
[30]	2012	Portugal	79, F	Compromised (diabetes mellitus)	Bilateral bullous keratopathy, rural area	Oral: V Intracamerular: F Topical: C, V	Yes	PK	Unrecovered (other causes: total retinal detachment)
[31]	2013	Italy	76, F	Compromised (local corticosteroid)	Corneal endothelial transplant (Fuchs’ dystrophy), rural area	Topical: A	Yes	Combined PK + PHACO	Recovered
[32]	2014	Japan	59, M	Competent	Farm worker	Oral: V Topical: Mi, N, V	No	Surgical debridement	Recovered (VA 20/100)
[33]	2014	South Korea	70, M	Competent	Unknown	Topical: A, N	No	Amniotic membrane	Recovered
[34]	2015	Japan	66, M	Compromised (local corticosteroid)	Bullous keratopathy	Topical: V	Yes	None	Recovered
[18]	2016	Spain	47, M	Competent	Agriculture worker, soft contact lens wearer	Oral: I, V Topical: N, V	Yes	Surgical debridement	Recovered
[35]	2020	USA (Minnesota)	65, M	Competent	Unknown	Intrastromal: A, M Topical: N, V Oral: A	No	None	Recovered
[36]	2021	Italy	85, M	Competent	Dirty gutter water	Topical: M, N, V	Yes	Surgical debridement, corneal patch	Recovered (patch after corneal perforation)
[37]	2021	Greece	84, M	Compromised (diabetes mellitus, local corticosteroid)	Agriculture worker, penetrating keratoplasty	Oral: P Topical: N, V	No	PK	Recovered (PK after corneal perforation)

Abbreviation: A: amphotericin B, C: clotrimazole, F: fluconazole, I: itraconazole, K: keratoconazole, M: miconazole, Mi: micafungin, N: natamycin, P: posaconazole, PK: perforating keratoplasty, PHACO: phacoemulsification, PPV: pars plana vitrectomy, Ref: reference, V: voriconazole.

## Data Availability

The data are available upon request.
